# Investigating Antimicrobial Resistance and ESBL Producing Gene in *Klebsiella* Isolates among Neonates and Adolescents in Southern Bangladesh

**DOI:** 10.1155/2022/7071009

**Published:** 2022-09-30

**Authors:** Afroza Akter Tanni, Nahid Sultana, Wazir Ahmed, Md. Mahbub Hasan, Md. Shakhawat Hossain, Sajjad Hossain Noyon, Md. Mobarok Hossain, Adnan Mannan

**Affiliations:** ^1^Department of Genetic Engineering & Biotechnology, Faculty of Biological Sciences, University of Chittagong, Chattogram-4331, Bangladesh; ^2^Disease Biology and Molecular Epidemiology Research Lab, Biotechnology Research & Innovation Centre (BRIC), Department of Genetic Engineering & Biotechnology, University of Chittagong, Chattogram, Bangladesh; ^3^Department of Microbiology, Chattogram Maa O Shishu Hospital, Agrabad, Chattogram, Bangladesh; ^4^Department of Neonatology, Chattogram Maa O Shishu Hospital, Agrabad, Chattogram, Bangladesh

## Abstract

**Background:**

Multidrug-resistant (MDR) clones of *Klebsiella pneumoniae* (*Kpn*) have been increasingly documented in community-acquired and nosocomial infections all around the globe. Extended-spectrum *β*-lactamases (ESBLs) are a rapidly evolving group of *β*-lactamase enzymes derived from SHV genes by mutations. This research work aimed to investigate and analyze the widespread prevalence of *Kpn* antibiotic resistance in different areas of the southern part of Bangladesh.

**Methods:**

This particular study was executed and implemented by using 501 clinical samples or isolates from two different hospitals in Chattogram. The disk diffusion method was used to detect *Kpn*'s sensitivity to 16 antibiotics in a drug susceptibility test. By using the PCR technique, the widespread prevalence of antibiotic-resistant gene bla_SHV-11_ was studied. Sequencing along with phylogenetic analysis was utilized to verify isolates with the bla_SHV-11_ gene.

**Results:**

Almost all of the *Kpn* isolates were spotted to be antibiotic-resistant. These *Kpn* isolates were resistant to *β*-lactams, aminoglycosides, and quinolones at high levels. The spatial analysis displayed that infections involving *Kpn* were more common in the urban areas (70%) than in the rural areas (30%). Neonates had substantially higher levels (*p* < 0.001) of resistance to multidrug than other age groups. Cefepime was identified as the most frequent antibiotic-resistant to all age groups (56.68%). The highest numbers of resistant isolates (36.92%) were found in urine samples. The ESBL gene bla_SHV-11_ was found in 38% isolates.

**Conclusion:**

The significant frequency of MDR *Kpn* harboring *β*-lactamases and AMR genes strongly suggests the requirement to develop effective antimicrobial resistance control and prevention measures in Bangladesh.

## 1. Introduction

Antimicrobial resistance (AMR) is a global burden affecting multiple sectors [1, 2]. Antibiotic resistance will cost the global economy $100 trillion by 2050, with a death rate of nearly 10 million people each year, surpassing cancer and heart disease [3, 4].

In Bangladesh, about 30–33% of MDR species such as ESKAPE (*Enterococcus faecium, Staphylococcus aureus, Klebsiella pneumoniae, Acinetobacter baumannii, Pseudomonas aeruginosa, and Enterobacter* spp.) pathogens were reported in 2015, and the rate steadily increased 2-fold during 2016–2018, peaking at around 62% in 2019 [5]. Because of a lack of awareness, resources, and nationwide monitoring, implementing current policies and initiatives for the issue of antibiotic resistance remains an obstacle. Bacterial genes that resist antibiotic action, known as resistance genes or “resistomes,” contribute to bacterial resistance against antibiotics on a global scale. Resistomes are highly widespread, tenacious, and can easily spread from a single type of bacterial strain to another belonging to a completely different genus through mechanisms such as gene transfer through horizontal techniques. As a result, antimicrobial resistance poses a worldwide concern [6, 7]. *Kpn *has been reported to be significantly resistant to the four main kinds of antibiotics, resulting in both MDR (multiple drug resistance) and XDR (extreme drug resistance) type *Kpn* development, as per the European Antimicrobial Resistance Surveillance Network [8].

Pandrug-resistant strains of carbapenemase resistant *Klebsiella pneumoniae* (CR-Kpn) are increasing (by about 14%) in Bangladesh [9]. A number of *Kpn*s isolated from clinical samples were resistant to third-generation cephalosporins (60.6% of cefpodoxime, 59.9% of ceftriaxone, and 52.1% of cefotiam). Along these lines, *Kpn* showed resistance to amoxicillin (91%) and nitrofurantoin (91%) in various recent studies [10, 11]. CR-Kpn strains and their relevant sequence types are continuously harboring a variety of resistant genes through transposable elements and horizontal gene transfer (HGT), the most common of which are New Delhi metallo-beta-lactamase (NDM), oxacillinase (OXA), and sulfhydryl variables (SHV-201, SHV-202) in different clinical sources [10, 12, 13]. As a result, treatment options are becoming increasingly limited.

The SHV-11 (sulfhydryl reagent variable) gene, which belongs to the ESBL genes' SHV-type variants, was demonstrated to have lactamase activity against beta-lactams such as penicillin and well-known third-generation antibiotic cephalosporins [11]. *Kpn* strains possessing the chromosomal SHV-11 *β*-lactamase gene are more likely than those carrying the chromosomal SHV-1 *β*-lactamase gene to generate the plasmid-mediated SHV-12 extended-spectrum lactamase [12]. The *β*-lactamase generating ESBL genes were detected in most of the multidrug-resistant isolates [13].

As a result of the fast appearance and spread of MDR superbugs, resistance to antibiotics in microorganisms has now become an impending global issue that can cause major damage. The objective of this research study was to determine the widespread presence and genetic changes of the gene that produces ESBL and the respective SHV-11 variant form in newborns and children (ages 0–18 years) infected with *Kpn* in Bangladesh. Understanding antibiotic resistance in commonly found microorganisms on a molecular level can provide opportunities for early management and implementation of proposed strategies before the consequences become too catastrophic.

## 2. Methods and Materials

### 2.1. Ethical Considerations

The institutional review board of Chattogram Maa Shishu O General Hospital Medical College approved the research study (CMOSHMC/IRB/2018/5). An oral consent was taken from the guardians of the patients. The need for written consent was waived by the ethics committee.

### 2.2. Study Setting

The research study was conducted at two hospitals of Chattogram between August 2018 to December 2019. Within the age cohort of ≤18 years old, a range of clinical samples was obtained from both outdoor and indoor *Kpn* patients. Patients admitted to hospitals were regarded as indoor patients, while those who visited hospitals but were not admitted were regarded as outdoor patients. Samples were collected from various hospital wards such as medicine, gynae, neonatal, and pediatric surgery, adult surgery, special care baby unit, neurology, diarrhoea, thalassemia, orthopedics, Intensive Care Unit (ICU), and Neonatal Intensive Care Unit (NICU). The demographic and epidemiological data of patients with *Kpn* infections were collected from medical records and microbiology databases owned and dispatched by the microbiology laboratory of Chattogram Maa-Shishu O General Hospital Microbiology lab and CHEVRON hospital and Clinical Laboratory (PTE) Ltd. The spatial map of the patient's address was created using layers available from the Bangladesh Geospatial Data Sharing Platform website (https://geodash.gov.bd/), and maps were created on the ArcGIS Desktop (Esri Inc., 109 Redlands, California, United States).

### 2.3. *Kpn* Strains and Phenotypic Tests

By following the CLSI (Clinical Laboratory Standard Institute) standards, culture characteristics, regular standard biochemical assays, and Gram stain methods were used to evaluate the *Kpn*-containing isolates. First, chocolate agar, Mac-Conkey agar medium, and blood agar were used to examine the shape as well as the colony of bacterial species. Then, gram stain methods were used to differentiate bacterial groups. After confirmation analysis of *Klebsiella pneumonia* (*Kpn*) by subculture method, a variety of standard biochemical tests were conducted (Voges Proskauer, indole, urease tests, TSI [triple sugar iron], motility, citrate, and methyl red) [14–16].

### 2.4. Antibiotic Susceptibility Test of *Kpn*

The CLSI-approved disc diffusion technique developed by Kirby and Bauer was usd to evaluate the antimicrobial susceptibility of *Kpn* isolates against 16 antimicrobial agents from seven different groups. An overnight culture of *Kpn* adjusted to standard suspension of isolate confirming 0.5 McFarland turbidity was inoculated on the surface of two Mueller Hinton agar (HIMEDIA, India) plates by using selective antibiotic disks, namely amikacin (30 *μ*g), amoxiclav (30 *μ*g), ampicillin (30 *μ*g), cefepime (30 *μ*g), cefixime (5 *μ*g), ceftazidime (30 *μ*g), ceftriaxone (25 *μ*g), chloramphenicol (30 *μ*g), ciprofloxacin, (5 *μ*g), cotrimoxazole (23.75 *μ*g), gentamicin (10 *μ*g), imipenem (10 *μ*g), levofloxacin (5 *μ*g), meropenem (24 *μ*g), netilmicin (10 *μ*g), and piperacillin (30 *μ*g) according to CLSI guidelines [17]. For negative control, a blank disk of filter paper was used. Multidrug-resistant samples were selected for molecular analysis. Isolates showing resistance to the highest number of antibiotic categories were chosen for further analysis. After incubation, the diameter of inhibition zones was measured, and the minimal inhibitory concentrations (MICs) were interpreted according to the CLSI standards [17]. Multidrug resistance (MDR) was calculated as per the standard definition by AP Magiorakos et al. 2021 and ESPAUR [18]. Here, MDR was defined as a *Kpn* isolate that was resistant to at least one antimicrobial agent from at least three antimicrobial classes.

### 2.5. Molecular Confirmation of blaSHV-11

For molecular identification of blaSHV-11, *Kpn* isolates' genomic DNA with the highest incidence of resistance to antibiotics was isolated by using the usual boiling technique [19, 20]. In a NanoDrop (Thermo Scientific) Spectrophotometer 2000, the concentration and purity of the isolated DNAs were evaluated at 260/280 absorbance. Polymerase chain reactions (PCR) were used to identify the blaSHV-11 gene from confirmed MDR Kpn isolate by using the primers listed below: Forward_5′-ATGCGTTATATTCGCCTGTGTATT-3′; Reverse_5′-GCGTTGCCAGTGCTCGATCAGCGC-3′ [21, 22] (annealing temperature 51.2°C) [21]. An entire amount of 25 *μ*L aliquot, where 12.5 *μ*L was a green master mix, 1 *μ*L Reverse and Forward primer each, 1 *μ*L Template DNA, and the rest was nuclease-free water, was set for PCR at 51.2°C by using a thermal cycler (Nyx technology).

### 2.6. Gel Electrophoresis

One gram agarose was dissolved into 100 ml of 1X (TAE) tris-acetate-ethylene-diamine-tetra-acetate for preparing a 1% (w/v) agarose gel. The agarose solution was heated in a microwave for around 3 minutes and then kept to be cooled to approximately 50°C. The molted transparent gel was poured into a casting tray having a comb set up and allowed to be semisolidified. The comb was removed after 20 minutes, and the tray holding the gel without the comb was kept in a gel electrophoresis chamber filled with TAE buffer, with 5 *μ*L of each amplified product inserted into a separate well of the agarose-made gel. A 5 *μ*L extended quick ladder and nuclease-free (NF) water as negative control were also added. For around 50 minutes, an electric current of 80 V and 400 mA (milli Ampere) was passed through the 1% gel electrophoresis chamber, after which the gel was submerged in an ethidium bromide-containing box for 30 minutes before being visualized and photographed by using UV light in the gel documentation chamber.

### 2.7. Sequence Analysis

The purified PCR products of bla_SHV-11_ genes were sequenced through the Sanger sequencing method. By using the BioEdit Program for sequence alignment (version 7.0.5.3), all raw sequences were combined to form a consensus. Following the merge, the sequence quality was checked through FastQC. Alignment and variation were validated by using BLASTn (https://blast.ncbi.nlm.nih.gov/Blast.cgi?PROGRAM=blastn&BLAST_SPEC=GeoBlast&PAGE_TYPE=BlastSearch) and BLASTp (https://blast.ncbi.nlm.nih.gov/Blast.cgi?PAGE=Proteins). All sequences were submitted to the database of NCBI after they were processed. MN551176, MN437452, MN551175, and MN551177 are the representing accession numbers for the SHV-11 gene-containing *Kpn* isolates.

### 2.8. Multiple Sequence Alignment (MSA) and Phylogenetic Tree Construction

To find further about *Kpn*'s evolutional history, 60 sequences of SHV-11 (source: biological and environmental samples) were extracted from pathogenwatch (as assembly files of *Kpn*) by filtering *Klebsiella* species from neighboring counties (Bangladesh, India, Myanmar, Pakistan, and Nepal). Prokka v1.14.5 was then used to annotate the assembly file in order to identify the blaSHV gene. After sorting the gene, Clustalomega v1.2.4 was used to align all the sequences, including our four sequences. Finally, iqtree v2.0.6 was used to generate a phylogenetic tree from the aligned sequences, and iTOL was used to annotate the tree.

### 2.9. Statistical Analysis

Epidemiological data were computed and sorted based on various categories in REDCap (http://data.dblab.org/redcap). The data were statistically evaluated by using IBM SPSS Statistical Software Version 25 (SPSS Inc., Chicago, IL, USA). Data belonging to the category type were contrasted by the two-tailed *χ*2 test or Fisher exact test, where *p* < 0.05 was taken as the level of significance. Categorical variables or qualitative data were presented as frequency or prevalence.

## 3. Results

### 3.1. Clinical Profile of Study Sample

A total of 501 strains were recovered from samples or isolates collected from both outdoor (51.89%, *n* = 260) and indoor (48.1%, *n* = 241) patients at twotertiary hospitals of Chattogram. Among the 501 participants, the overwhelming majority of the patients had been in the medicine (15.77%, *n* = 41) ward; followed by the neonatal (12.69%, *n* = 33), pediatric (11.9%, *n* = 31), and general floor (11.15%, *n* = 29) wards. Bulk of the *Kpn* isolates were procured from urine (36.9%, *n* = 185) samples, followed by tracheal aspirate (19.16%, *n* = 96), pus (11.97%, *n* = 60), blood (17.56%, *n* = 88), sputum (5.38%, *n* = 27), wound swab (4.99%, *n* = 25), HVS (2.39%, *n* = 12), throat swab (1.19%, *n* = 6), and umbilical swab (0.39%, *n* = (2) (Table 1). The following were the MDR prevalence rates found in tracheal aspiration, urine, blood, pus, sputum, throat swab, HVS, wound swab, and umbilical swab samples: 84.4%, 67.56%, 79.55%, 68.33%, 40.74%, 83.33%, 41.67%, 50%, 40.74%, 68%, and 100%, respectively.

**Figure 1:**
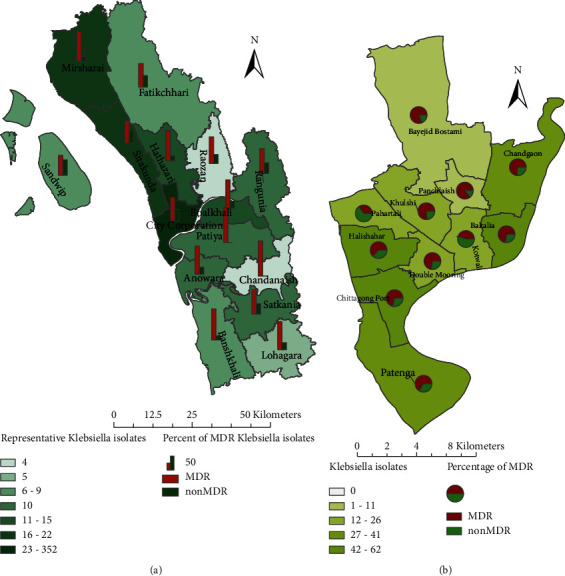
Spatial distribution of antibiotic resistance of Kpn isolates among the patients of Chattogram. (a) Rural area; (b) Urban area (Chittagong City Corporation).

The spatial analysis revealed that 70% (*N* = 352) of the *Kpn* infections originated from the Chattogram City Corporation area, while 30% (N=149) were from other Upazila of the Chattogram District. MDR prevalent *Kpn* pathogens (acquired resistance to at least two drugs from 1 belonging to the 7 groups of antibiotics and 1 belonging to the other 6 groups of antimicrobial agent classes) seemed to be higher in the city area (79%) than in the noncity corporation area (68%) with a 56% (Sandwip Upazila) to 100% (Chandanaish and Patiya Upazila) range (Figure 1(a)). Within the city corporation, the lowest burden of MDR was uncovered in the Pahartali and Kotwali areas, with a percentage of 35% and 45%, respectively. The highest burden was encountered in the Panchlaish area (86%) (Figure 1(b)).

### 3.2. Pattern of Drug Resistance

**Figure 2:**
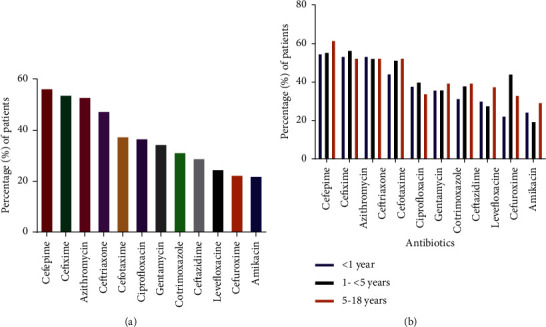
Response to different antibiotics regardless of the age, gender, or sample type of patients. (a) Percentages of resistance against various antibiotics among different patients; (b) antibiotic resistance among different age groups.

MDR was encountered in 71.06% of individuals infected with *Kpn* (Table 1). The bulk of the sample isolates were detected to be resistant to fourth-generation cephalosporin, namely cefepime (56.68%), third-generation cephalosporins, namely cefixime (54.09%), ceftriaxone (47.70%), cefotaxime (37.72%), and ceftazidime (29.14%); azithromycin (Macrolide) (53.29%), and gentamicin (Aminoglycoside) (34.73%) (Figure 2). Few isolates were found to be sensitive to cefuroxime (22.55%) and imipenem (8.58%).

### 3.3. Identification of blaSHV-11 Gene in *Kpn* Isolates

**Figure 3:**
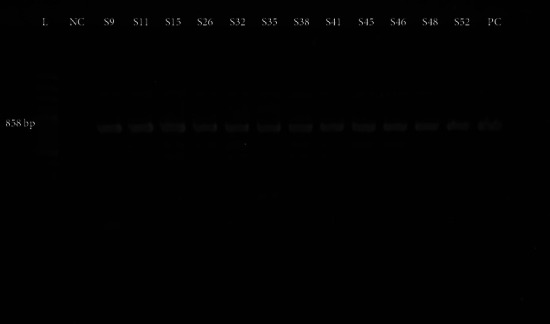
Representative gel image showing amplification of *bla*_*SHV-11*_ gene (858 bp): Lane L Ladder (100 bp); Lane NC: negative control; Lane PC: positive control; SHV-11 positive *Kpn* isolates; Lane S9– S52: clinical isolates.

The extended-spectrum *β*-lactamase blaSHV-11 gene, with a particular product size of 858 bp, was found in 38% isolates (Figure 3). Infection rates were higher in males (52.6%) than females (47.4%). The blaSHV-11 gene was identified significantly (*p* < 0.05) more in urine samples at a substantially higher rate of 34.2% than in the other samples. Also, this was found in the tracheal aspiration (18.4%), blood (13.2%), pus (15.8%), sputum (7.9%), as well as wound swab (10.5%). The blaSHV-11 gene was identified in 60.5% of indoor patients and 39.5% of outdoor patients. Among them, 71.92% and 68.05% were MDR, respectively.

### 3.4. Phylogenetic Analysis and Evolutionary Relationships of Taxa

**Figure 4:**
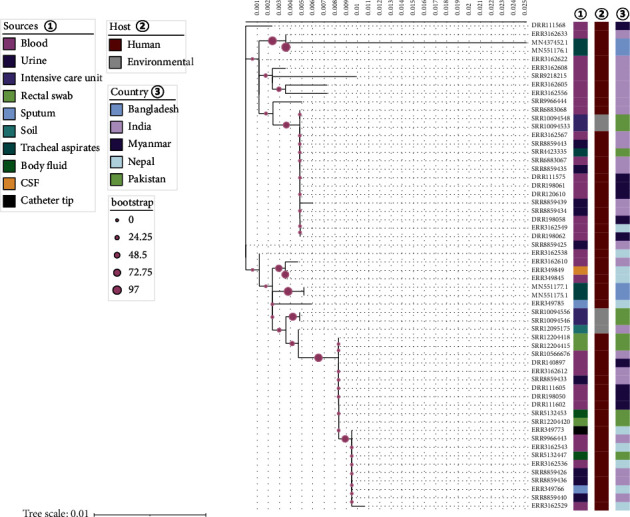
Molecular Phylogenetic analysis of SHV-11 protein. The evolutionary history was inferred by using the maximum likelihood method based on the HKY model. Here, the tree was built with the bootstrap value (100). The tree was drawn to scale, with branch lengths measured in the number of substitutions per site.

By observing the tree, it became obvious that two of our sequences (MN551177.1 and MN551175.1) had significant variations (Figure 4). For that reason, they were observed to be clustered with each other. Though the other two *Kpn* sequences of this study were also observed to be clustered together, there was a minor variation between them. The source of each gene was enclosed in Supplementary Table 1, including accession ID, isolation source, year, and host. This study thus illustrated changes among all the sequences, but it needs further study with more sequences from Bangladesh to understand clustering in depth.

## 4. Discussion

In recent times, *Kpn* has emerged and spread as an opportunistic, drug-resistant pathogen, creating grave clinical concern [22]. Its prevalence has expanded to a variety of niches, from mucosal layers of animals and humans to water from sewerage channels, soil, and even vegetation [22, 23]. Similarities in biochemical properties, virulence, and pathogenicity were noticed in environmental origin and clinical origin-based *Kpn* strains [22, 23]. In terms of antibiotic resistance, clinical *Kpn* strains have been determined to be more resistant to several types of antibiotics than those found in the environment [24].

First of all, a higher number of resistant strains were recovered from the specimens that were taken from urban patients than from rural ones. Secondly, since the number of pharmacies in the urban areas is higher than that in the rural ones, the random use of antibiotics is significantly rising there. The pattern is also extending into the aquatic and food chains. As a result, secondary infections brought on by resistant types of bacterial strains are also occurring more frequently. Because of data gaps and subpar medical care, the genuine reality of rural areas is not revealed here. *Kpn* infection rates were noticeable in both females and males in this particular research work, especially with multidrug-resistant strains of *Kpn*. *Kpn *isolates are often the most encountered pathogen responsible for acute pyelonephritis during childhood [25]. However, Kpn causes pyelonephritis in females and upper UTI in males >20 years old [26].

Among the various age groups considered for the research work, toddlers (12 months-36 months) exhibited the highest MDR (81.16%), followed by adolescents (12–18 years) (73.33%). MDR was spotted in all other age groups, indicating a significant prevalence of resistance to antimicrobial drugs in children and neonates in general. These data appear to support the general consensus that resistance to antimicrobial drugs is on the rise both nationally and globally [27, 28]. The widespread usage of antimicrobial drugs, along with an undeveloped immune system in children and neonates, creating selective pressure, appears to be the major cause of MDR bacterial strains comparable to *Kpn* infections in Bangladesh. Poor hygiene practice, sanitation, and subpar infection control among children and neonates in healthcare settings are some of Bangladesh's most likely reasons for antibiotic resistance [29].

*Kpn* infection rates among neonates and children are noticeably high, as seen in this current study. In hospitals, children with malnourished conditions have higher antibiotic resistance for infections such as *Kpn* infection due to dehydration, acute wasting, etc. These health conditions among children, neonates, and adolescents alter gut functions and damage mucosal levels which makes it easy for *Kpn* to translocate to other places, for example, the kidneys from the gut [30]. In Bangladesh, hospitals experience high numbers of neonates, child patients with malnutrition diseases, and infections of the kidneys or the urinary tract, which make it easier for *Kpn* along with other bacteria, for example, *E. coli,* to spread internally and cause community infections [31].

In this work, higher infection rates and resistance rates were observed among patients in hospitals (60.5%) which may indicate hospital-acquired resistance to antimicrobial drugs among patients (Table 1). Prior research work found that *Kpn* samples recovered from clinical equipment, hands/clothes of medical staff, and clinical samples from the hospital, such as the blood and urine, were more resistant than the general rate [32]. The high mutation rate of *Kpn* along with its fast spread among communities over time could be a reason for hospitalized patients having bacterial strains with heightened levels of MDR. Studies also support that the colonization rate of *Kpn* was revealed to have increased in patients in direct proportion to the duration of their hospital stay. Rates of colonization in hospital-staying patients were four times higher in carriers in comparison to noncarriers of bacterial infection [23].

*Kpn* spreads throughout the body from the gut in both adults and children. It is found in sputum and urine samples because these samples come from areas where frequent bacterial turnover occurs, such as kidney infections or UTIs [33]. Mechanisms used by transposable elements and plasmids of *Kpn* strains help it to spread and cause infections such as nosocomial pneumonia and UTI [34].

In our research work, MDR was exhibited in the majority (70%) of the *Kpn* sample isolates. We also identified that these isolated samples were not susceptible or rather greatly resistant to common antibiotics used either singularly or in association with other drugs to treat *Kpn* infections, such as *β*-lactam antibiotics- 2nd and 3rd generation of cephalosporin, aminoglycosides, macrolides, and quinolones (Figure 2). Causes and risk elements that support this pattern of resistance to antimicrobial drugs include gene transfer through a horizontal method, incomplete cycles of medicine intake, taking nonprescribed antibiotics, limited diagnostic facilities, limited monitoring and surveillance systems for patients, immunocompromisation, rapid community infections, and antibiotic overuse in organic produce [17, 35–37].

*Kpn'*s spatial distribution among neonates and young adults in our work suggests that MDR is prevalent in this population in both rural and urban regions (Figure 3). MDR infections account for around 70% of all *Kpn* infections. When compared to equivalent figures in a developed country, this is extremely high. According to one particular recent research from Public Health England, around 21% of patients suffering from bloodstream infections are diagnosed with resistant key pathogens [38]. During 2015 and 2019, there was a 32.5% elevation in resistant infections, as reported by the same report. However, since our study depicts cross-sectional data for the *Kpn* mediated MDR burden in Chattogram only, further conduction of a systematic study on a larger sample and location span is necessary to appraise the actual burden of resistant pathogens and find out the cause to further develop a sustainable mitigation plan to tackle the MDR related complications.

In this research work, several clinical isolates (38%) were SHV-11 positive, showing a highly resistant pattern to the bulk of the clinically used antibiotics. Neonates and infants were most prone to infection. Previously, this gene was reported in water samples, drain water, and bed trails from the environment [39]. In a previous study, in MDR, *Kpn* isolates SHV-12 were discovered from surface water [40]. Prolonged hospital stay, ICU, and invasive positive ESBL isolate input through central venous, urinary, and endotracheal catheters are all risk factors for ESBL-containing isolate dissemination [41].

From the evolutionary tree, it is apparent that MN551177 is one of the most ancient genes of *Kpn* species (Figure 4). It shared a common ancestry with a strain of accession number HBL89233.1. Due to the high sequence conservancy of the recently discovered gene mutation of SHV-11 in Chittagong, MN551175, MN551177, and MNMN437452 showed a cluster in the tree. Yet, the first two of these clusters are of very common ancestor on account of their own sequence in two directions, AVO16696.1.

Bangladesh is particularly vulnerable to the ramifications of AMR and the dissemination of MDR pathogens due to a lack of knowledge about the proper use and dose of various antibiotics. This included selling off and taking prescription antibiotics without a physician's advice, antimicrobial drug usage in cattle feed and not completing a full cycle of antibiotic dosage [42–44]. Throughout the country, antimicrobial drugs are readily accessible and can usually be taken without a proper prescription, resulting in a spiral in their irrational use across industries, resulting in pollution and uncontrollable antimicrobial resistance dissemination [40, 42, 45, 46].

Although the results obtained from this study are substantial, it should be taken into account that the study was conducted with a limited number of samples and was solely based on patients from two hospitals in southern Bangladesh who were unable to exhibit the complete picture of the whole country. Despite these limitations, this study will serve as a benchmark for further work on antibiotic resistance among neonates and children in Bangladesh.

## 5. Conclusion

In this study, significant prevalence of *Kpn* isolates resistant to antimicrobial drugs was observed among neonates and children. Moreover, high occurrence of *Kpn* strains with a considerable distribution of antibiotic-resistant genes was reported. Existence of the ESBL-producing gene, and an elevated persistence of antimicrobial drug resistance to various antibiotic classes, raise a serious public and community health concern regarding the worsening condition of hospital infections. To be able to fully assess the epidemiological implications of *Kpn* strains and their effects, it is high time to consider conducting molecular studies on larger study samples. Identifying the prevalence of virulence factors in various strains of *Kpn* will help to design new therapeutic option and implement more efficient policies for preventing a future pandemic related to superbugs.

## Figures and Tables

**Table 1:** Clinical characteristics of *Kpn* infected patients (*n* = 501).

Variables	Resistant to one/two antibiotic	Multidrug-resistant	Total cases (*n* = 501)	Pearson's c2	*P*-value
*Gender*					
Male	36 (14.87%)	189 (78.099%)	242 (48.303%)	104.04	<0.001
Female	75 (28.95%)	167 (64.478%)	259 (51.696%)	34.975	

*Age Groups*					
Baby/Newborn (0–4 weeks)	45 (20.93%)	143 (66.51%)	215 (42.91%)^*∗*^	51.085	<0.001
Baby/Infant (4 week–<1 year)	18 (22.22%)	58 (71.604%)	81 (16.167%)	21.053	
Toddler (1– <3 years)	8 (11.59%)	58 (84.057%)^*∗*^	69 (13.77%)	37.879	
Preschooler (3– <5 years)	11 (39.285%)	17 (60.71%)	28 (5.588%)	1.2857	
Childhood (5– <12 years)	15 (23.809%)	46 (73.015%)	63 (12.57%)	15	
Adolescent (12–18 years)	8 (17.77%)	33 (73.33%)	45 (8.98%)	15.244	

*Patient Type*					
Indoor/Ward	51 (19.615%)	193 (74.23%)^*∗*^	260 (51.896%)	82.639	
Outdoor	60 (24.89%)	163 (67.634%)	241 (48.104%)	47.574	

*Sample Types*					
Urine	49 (26.486%)	125 (67.567%)	185 (36.92%)^*∗*^	33.195	<0.001
Tracheal aspirates	13 (13.54%)	81 (84.375%)^*∗*^	96 (19.16%)	49.191	
Pus	17 (28.33%)	41 (68.33%)	60 (11.976%)	9.931	
Blood	9 (10.227%)	70 (79.545%)	88 (17.56%)	47.101	
Sputum	10 (37.037%)	11 (40.740%)	27 (5.389%)	0.047619	
Wound swab	6 (35.294%)	17 (68%)	25 (4.99%)	5.2609	
HVS	7 (58.33%)^*∗*^	5 (41.6666%)	12 (2.395%)	0.33333	
Throat swab	0 (0.00%)	5 (83.33%)	6 (1.197%)	5^*∗*^	
Umbilical swab	0 (0.00%)	2 (100%)	2 (0.399%)	2^*∗*^	

*Regional Distribution*					
Urban	137	178	315	0.69	0.4
Rural	73	113	186		

^*∗*^Correlation analysis between single drug and multidrug-resistance among different groups of *Kpn* isolates. ^*∗*^ indicates statistically significant (*P* value is less than 0.05).

## Data Availability

The data used to support the findings of this study are included within the article.

## Supplementary Materials

Supplementary Materials Supplementary Table 1. Information of the sequences of *Kpn* infected patients (n = 60).
