# Supplementary material for: Investigating Antimicrobial Resistance and ESBL Producing Gene in Klebsiella Isolates among Neonates and Adolescents in Southern Bangladesh
Source: Can J Infect Dis Med Microbiol. 2022 Sep 30;2022:7071009. doi: 10.1155/2022/7071009 (PMC9553706; doi:10.1155/2022/7071009)
Supplement: Supplementary Materials — Supplementary Table 1. Information of the sequences of Kpn infected patients (n = 60). [file 7071009.f1.docx]

**Supplementary file**

**Supplementary Table-1: Information of the sequences of** *Kpn* (n = 60)

| **Accession ID** | **year** | **Country** | **Isolation source** | **Host** |
| --- | --- | --- | --- | --- |
| ERR349766 | 2012 | Nepal | Sputum | Human |
| ERR349773 | 2012 | Nepal | Catheter tip | Human |
| ERR349785 | 2012 | Nepal | Sputum | Human |
| ERR349845 | 2012 | Nepal | Blood | Human |
| ERR349849 | 2012 | Nepal | CSF | Human |
| SRR5132447 | 2013 | Pakistan | Body fluid | Human |
| SRR5132453 | 2013 | Pakistan | Body fluid | Human |
| ERR3162529 | 2013 | Nepal | Blood | Human |
| SRR9966444 | 2013 | India | Blood | Human |
| SRR9966443 | 2014 | India | Blood | Human |
| SRR4423335 | 2015 | Pakistan | Tracheal aspirates | Human |
| DRR111568 | 2015 | Myanmar | Blood | Human |
| SRR6883067 | 2015 | India | Blood | Human |
| SRR6883068 | 2015 | India | Blood | Human |
| SRR10094533 | 2016 | Pakistan | Intensive care unit | Environmental |
| SRR10094546 | 2016 | Pakistan | Intensive care unit | Environmental |
| SRR10094548 | 2016 | Pakistan | Intensive care unit | Environmental |
| SRR10094556 | 2016 | Pakistan | Intensive care unit | Environmental |
| ERR3162536 | 2016 | Nepal | Blood | Human |
| ERR3162538 | 2016 | Nepal | Blood | Human |
| ERR3162543 | 2016 | Nepal | Blood | Human |
| ERR3162549 | 2016 | Nepal | Blood | Human |
| DRR111575 | 2016 | Myanmar | Blood | Human |
| DRR111602 | 2016 | Myanmar | Blood | Human |
| DRR111605 | 2016 | Myanmar | Blood | Human |
| DRR140897 | 2016 | Myanmar | Blood | Human |
| ERR3162624 | 2016 | India | Blood | Human |
| SRR10566676 | 2016 | India | Blood | Human |
| SRR9218215 | 2016 | India | Blood | Human |
| DRR120610 | 2017 | Myanmar | Blood | Human |
| DRR198050 | 2017 | Myanmar | Blood | Human |
| DRR198058 | 2017 | Myanmar | Blood | Human |
| DRR198061 | 2017 | Myanmar | Blood | Human |
| DRR198062 | 2017 | Myanmar | Blood | Human |
| ERR3162556 | 2017 | India | Blood | Human |
| ERR3162567 | 2017 | India | Blood | Human |
| ERR3162605 | 2017 | India | Blood | Human |
| ERR3162608 | 2017 | India | Blood | Human |
| ERR3162610 | 2017 | India | Blood | Human |
| ERR3162612 | 2017 | India | Blood | Human |
| ERR3162622 | 2017 | India | Blood | Human |
| ERR3162633 | 2017 | India | Blood | Human |
| SRR8859425 | 2017 | India | Urine | Human |
| SRR8859426 | 2017 | India | Urine | Human |
| SRR8859433 | 2017 | India | Urine | Human |
| SRR8859434 | 2017 | India | Urine | Human |
| SRR8859435 | 2017 | India | Urine | Human |
| SRR8859436 | 2017 | India | Urine | Human |
| SRR8859439 | 2017 | India | Urine | Human |
| SRR8859440 | 2017 | India | Urine | Human |
| SRR8859443 | 2017 | India | Urine | Human |
| SRR12095175 | 2018 | India | Soil | Environmental |
| SRR12204415 | 2019 | Pakistan | Rectal swab | Human |
| SRR12204418 | 2019 | Pakistan | Rectal swab | Human |
| SRR12204420 | 2019 | Pakistan | Rectal swab | Human |
| MN551176.1 | 2019 | Bangladesh | Tracheal aspirates | Human |
| MN437452.1 | 2019 | Bangladesh | Tracheal aspirates | Human |
| MN551175.1 | 2019 | Bangladesh | Tracheal aspirates | Human |
| MN551177.1 | 2019 | Bangladesh | Tracheal aspirates | Human |
